# Insulin/IGF Axis in Breast Cancer: Clinical Evidence and Translational Insights

**DOI:** 10.3390/biom11010125

**Published:** 2021-01-19

**Authors:** Federica Biello, Francesca Platini, Francesca D’Avanzo, Carlo Cattrini, Alessia Mennitto, Silvia Genestroni, Veronica Martini, Paolo Marzullo, Gianluca Aimaretti, Alessandra Gennari

**Affiliations:** 1 Department of Translational Medicine, University of Eastern Piedmont, Via Solaroli 17, 28100 Novara, Italy; paolo.marzullo@med.uniupo.it (P.M.); gianluca.aimaretti@med.uniupo.it (G.A.); alessandra.gennari@med.uniupo.it (A.G.); 2 Division of Oncology, University Hospital “Maggiore della Carità”, 28100 Novara, Italy; francesca.platini@maggioreosp.novara.it (F.P.); francesca.davanzo@maggioreosp.novara.it (F.D.); carlo.cattrini@maggioreosp.novara.it (C.C.); alessia.mennitto@maggioreosp.novara.it (A.M.); silvia.genestroni@maggioreosp.novara.it (S.G.); veronica.martini@uniupo.it (V.M.); 3 Lab of Immuno-Oncology, CAAD, Center of Autoimmune and Allergic Disease, University of Eastern Piedmont, 28100 Novara, Italy; 4 Division of General Medicine, IRCCS Istituto Auxologico Italiano, Ospedale S. Giuseppe, 28921 Piancavallo-Verbania, Italy

**Keywords:** breast cancer, host metabolism, insulin resistance, IGF, BMI

## Abstract

Background: Breast cancer (BC) is the most common neoplasm in women. Many clinical and preclinical studies investigated the possible relationship between host metabolism and BC. Significant differences among BC subtypes have been reported for glucose metabolism. Insulin can promote tumorigenesis through a direct effect on epithelial tissues or indirectly by affecting the levels of other modulators, such as the insulin-like growth factor (IGF) family of receptors, sex hormones, and adipokines. The potential anti-cancer activity of metformin is based on two principal effects: first, its capacity for lowering circulating insulin levels with indirect endocrine effects that may impact on tumor cell proliferation; second, its direct influence on many pro-cancer signaling pathways that are key drivers of BC aggressiveness. Methods: In the present review, the interaction between BC, host metabolism, and patients’ prognosis has been reviewed across available literature evidence. Conclusions: Obesity, metabolic syndrome, and insulin resistance are all involved in BC growth and could have a relevant impact on prognosis. All these factors act through a pro-inflammatory state, mediated by cytokines originated in fat tissue, and seem to be related to a higher risk of BC development and worse prognosis.

## 1. Introduction

In Western Countries, a large proportion of diseases, including cancer, can be attributed to a Western lifestyle, in association with physical inactivity and metabolic impairment. A growing body of evidence suggests a strong relationship among type 2 diabetes mellitus (T2DM), insulin, obesity, and cancer. The association between insulin and cancer is based on the strong anabolic effect of hyperinsulinemia that leads to proliferative tissue abnormalities, stimulation of DNA synthesis, and cell proliferation [1].

Preclinical studies demonstrated that insulin can promote tumorigenesis through a direct effect on epithelial tissues or indirectly by affecting the levels of other modulators, such as the insulin-like growth factor (IGF) family of receptors, sex hormones, and adipokines [2]. IGFs are endocrine mediators of growth hormones that act in a paracrine and autocrine fashion to regulate cell growth, differentiation, apoptosis, and transformation in different tissues, including breast tissue. Insulin receptors (IR) regulate glucose, protein, and lipid metabolism through the phosphatidylinositol 3-kinase (PI3K)/AKT pathway and through the activation of the RAS/RAF/MAPK kinase/ERK cascade that is involved in cell proliferation, survival, and migration [3].

Breast cancer (BC) is the most common cancer in women, with over 2 million of new diagnosis/year in 2018 in the world [4]. In the last years, the possible interactive role of host metabolism and BC has risen in importance, and many clinical and preclinical studies have been developed to understand their possible correlations. In particular, obesity has been consistently reported as a risk factor for different BC subtypes, including triple negative (TNBC) and luminal BC [5,6]. Available evidence also shows that BC cells have significantly higher insulin receptor (IR) levels than normal breast cells [7], suggesting that this signaling pathway may play a role in BC development and progression, and may become a potential therapeutic target. Furthermore, epidemiologic studies have shown that high levels of plasmatic IGF are correlated with an increased risk of several cancers, including breast, and that IGF-2 signaling is possibly associated with cancer progression [8]; it is also known that the loss of tumor suppressor genes such as BRCA1, p53, and PTEN leads to an increase in IGF-1R expression in tumors [9].

In this preclinical context, E-cadherin is a putative regulator of IGF1 signaling; the loss of E-cadherin expression can directly increase IGF1-R pathway activation in many BCs. The oncogenic signaling network may be influenced by the regulation of IGF1-R by E-cadherin, particularly because E-cadherin itself is involved in mechanisms of escape of tumor cells and metastasization. In addition, the transcription of E-cadherin is repressed or genetically lost in subset of breast tumors, such as invasive lobular breast carcinoma (ILC), in which its loss of functional occurs in about 95%. Due to the loss of E-cadherin protein, ILC cells grow in linear patterns throughout the breast tissue, lacking the ability to form adhesion junctions, in contrast to the solid mass growth of the most frequent subtype of breast cancer, invasive ductal breast carcinoma (IDC) [10,11].

In BC patients, the possible role of the IGF pathway in resistance to cancer therapy has been investigated, although the exact mechanism is not completely clear. In particular, the IGF pathway seems to be involved in modulating the expression of Epidermal Growth Factor receptors (EGFR) in cancer cells, and, as a consequence, it might have a key role in inducing resistance to endocrine therapies in estrogen receptor (ER)-positive BC patients [12,13]; in addition, direct interactions between the IGF-1R and the HER2 pathway have been reported and may contribute to the resistance to anti-HER2–targeted therapy [14] (Table 1).

## 2. Host Metabolism and Breast Cancer Risk

The possible relationship between cancer and obesity is very complex: Body mass index (BMI), an easy and immediate biometric index, deriving from weight and height and routinely used in clinical practice, cannot be considered an exact index of metabolic health, as obese patients can be metabolically healthy and normal-weight patients can be un-healthy. Moreover, its role as a risk factor for BC is dual: recent evidence has shown that increased adiposity in childhood and in early adulthood is associated with a reduced risk of BC, whereas increased adiposity after menopause is associated with an increased BC risk [17]. A large pooled analysis on about 750,000 premenopausal women demonstrated a higher risk of BC in those who were underweight as compared to those obese, showing an inverse association between BMI and BC risk, suggesting a critical correlation between young adulthood adiposity and cancer; this association seems to be independent from other well-known BC risk factors. The association between BMI and hormone receptor positive BC is stronger in young adulthood, but not consistent in later ages, while the correlation between obesity and TNBC is more controversial.

The association between estrogen receptor-positive (ER+) BC and obesity identifies a possible hormonal role in tumorigenesis, as the adipose tissue is the principal site of estrogen release in childhood; for this reason, overweight in childhood seems to induce early breast differentiation and to increase expression of tumor suppression gene [18].

The relationship between BC subgroups and obesity has been investigated in an Italian trial on 2148 women. Overweight/obese women had significantly larger tumors at diagnosis than under/normal weight women, both in pre-menopause and post-menopause. Postmenopausal women with BMI > 25 had a significantly higher percentage of hormone positive tumors than lean women, probably because insulin itself stimulates ovarian synthesis of androgens, induces expression of growth hormone (GH) receptors, decreases liver production of sex hormone binding globulin (SHBG) and two IGF-binding proteins (IGFBP-1 and -2), increasing the bio-availability of sexual hormones and IGF-1 [19].

In postmenopausal women, the presence of metabolic disorders and obesity has been consistently identified as a strong risk factor for BC [20], possibly due to the underlying chronic inflammation at the level of peripheral tissues [21]; furthermore, hyperinsulinemia itself was found to be an independent risk factor for post-menopausal BC [22]. In particular, the most striking evidence is reported in the Nurses’ Health Study, which included 5204 women diagnosed with BC between 1976 and 2000. In this study, women experiencing a gain in BMI during the observation period, ranging from 0.5 to 2 kg/m^2^ or >2 kg/m^2^ had a relative risk of BC increased by 1.35 and 1.64 times, respectively [23].

Association between obesity and BC seems also to be modulated by fat distribution: in post-menopausal patients, an increased fat mass of the trunk is related with a higher risk of BC [21,24]. Indeed, post-menopausal women tend to experience a re-distribution of body fat from a gynoid to an android pattern, which is supposed to be more metabolically important than general obesity and is also linked to the occurrence of the metabolic syndrome and cardiovascular disease [25].

An immunohistochemical analysis of levels of IGFR-1 was performed in a group of 144 females with TNBC to evaluate a possible correlation with their prognosis. Immunohistochemistry was performed on paraffin-embedded tumor tissues: An increase in IGFR-1 expression (99%) was detected in the majority of cases (63%), associated with reduced overall survival (OS) and disease free survival (DFS) [26].

While the relationship between BMI and ER + BC is well established, available evidence about the possible association between TNBC and BMI is less clear [27,28].

A pooled analysis on the Breast Cancer Association Consortium revealed that among women <50 years of age, obesity was associated more frequently with hormone negative BC than with hormone positive tumors and, by including human epidermal growth factor receptor 2 (HER2) receptors, obesity was confined to TNBC [29].

## 3. Host Metabolism and Breast Cancer Prognosis

The association between BMI and BC prognosis is supported by several observations [30]. Obesity represents a well-known pro-inflammatory state, resulting in increased levels of inflammatory cytokines such as TNFα and IL-6 [31]. TNF-α is a well-documented pro-inflammatory cytokine that is up-regulated in BC and associated with BC recurrence. Similarly, high circulating IL-6 levels are correlated with a poor prognosis in BC patients [32]. Indeed, elevated systemic levels of this cytokine are a biomarker of tumor burden and could be found in cases of impaired metabolism and physical inactivity [33].

The possible role of insulin resistance has been evaluated in a trial including 55 female patients with operable stage III BC. Thirteen (54.5%) patients had insulin resistance associated with a higher BMI, triglyceride, and fasting glucose level compared to patients without insulin resistance. As a result of this study, the probability of having a pathological complete response (pCR) after neo-adjuvant therapy in patients with insulin resistance was lower compared to those without insulin resistance, suggesting a possible negative effect of insulin resistance on pCR following neoadjuvant therapy, particularly with hormone-positive and Her-2 negative cases of non-diabetic BC [34].

Recently, the role of BMI on BC prognosis has been evaluated in 1066 women with high-risk early BC enrolled in a phase III trial of adjuvant chemotherapy (IBIS). In this specific population, there was no evidence of a detrimental effect of increased BMI, suggesting that in the presence of aggressive biological subtypes, host factors might have a minor effect on patients’ prognosis [35].

Epidemiologic evidences suggest that obesity worsens BC prognosis due to the increase of recurrence, metastasis, and mortality. As previously mentioned, in overweight or obese patients, BCs tend to be bigger at the diagnosis, with positive lymph-nodes, and higher tumor burden and grade [36].

In advanced BC disease, obesity is also related to a worse impact in response to chemotherapy, which dosage could often be reduced to avoid toxicities; furthermore, obesity increases risks of every treatment complication and is associated to an incidence of metastatic BC disease regardless of tumor subtype [37].

A systematic literature review and meta-analysis of follow-up studies by Chan et al. clearly supports that, in breast cancer survivors, higher BMI is consistently associated with lower OS. This increased risk is similar in pre- or post-menopausal BC: furthermore, obesity has a protective effect in pre-menopausal women on the development of BC, whereas it has no protective effect on survival after pre- menopausal BC. Compared with normal weight women, significant or borderline significant increased risks of BC mortality were only observed for morbidly obese (≥40 kg/m^2^) women and not for women in other obesity categories [38].

The relationship between BMI in BC growth seems to be mediated by hyperinsulinemia. There is evidence that metabolic changes associated with insulin resistance are involved in tumor development and aggressiveness. Diabetes has also been associated with a more aggressive cancer phenotype and a poorer response to treatment, worsening its prognosis [39].

In literature data, obesity tends to be associated with a risk reduction of BC among pre-menopausal women, that may be attributed to lower levels of serum estradiol and progesterone. Conversely, obesity is associated with an increased risk of BC in postmenopausal women, related to higher levels of circulating bioavailable estrogen [40].

To explore the association between BC and risk and prognostic factors related to body size, the CASH study (cancer and steroid hormones) has been conducted on 3924 women: in this cohort, adult BMI was significantly associated with BC mortality, in accordance with a growing body of evidence suggesting that obesity could be an independent and preventing risk and prognostic factor in BC [41].

Insulin resistance has been shown to be associated with the luminal B BC subtype in a cohort of 760 post-menopausal patients. The multivariate analyses showed that an Homeostatic model assessment index (HOMA-I) ≥2.0 was significantly correlated with luminal B/HER-2-negative subtype. Moreover, Ki-67 was higher in insulin resistant patients [42].

Potential association with obesity, metabolic syndrome, and insulin resistance was deeply investigated also for TNBC. The IGF-1 receptor is expressed also on the surface of TNBC cells; its signal transduction can increase cell proliferation and promote cell survival in vitro in several TNBC cell lines. Conversely, decreased IGF-1 receptor expression seems to reduce cellular proliferation [43].

A pooled analysis revealed that association between obesity and metabolic syndrome is more frequently observed in TNBC vs. ER+/HER2 tumors, particularly in younger women (age < 50) [29]. Moreover, women with triple-negative tumors seem to have a high prevalence of metabolic syndrome, characterized by increased BMI and circulating levels of insulin and related growth factors, leading to uncontrolled cell proliferation [44] and worse prognosis. Several other studies were conducted to investigate the potential role of obesity in TNBC development, with controversial results [45].

In conclusion, obesity, metabolic syndrome, and insulin resistance are mutually involved in BC prognosis. All these factors seem to act through a pro-inflammatory state, mediated by cytokines originating in the adipose tissue. In particular, android fat pattern is related to a higher risk of BC and a worse prognosis.

## 4. The Role of Metformin

Metformin is an oral biguanide that inhibits hepatic gluconeogenesis and sensitizes insulin action at the peripheral level, and it is one of the drugs of choice for the management of T2DM [46]. It inhibits hepatic glucose production through an LKB1/AMPK-mediated mechanism; it also improves insulin sensitivity in peripheral tissues and reduces the incidence of diabetes in persons at high risk, with beneficial effects persisting for at least 10 years [47]. It has a good safety profile and an extremely low cost, thus being easily accessible in clinical practice.

**Figure 1:**
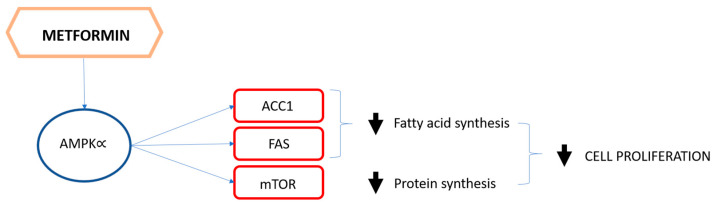
The pathway that corroborates the hypothesis of the direct effect of metformin on tumor cell proliferation.

**Figure 2:**
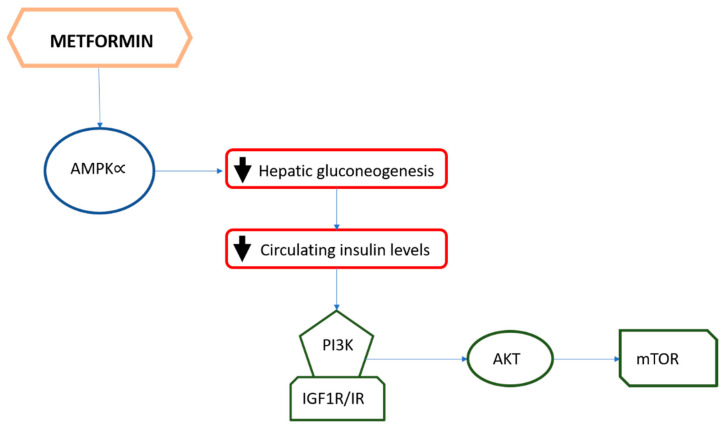
The pathway involved in the indirect effect of metformin in inhibition of cancel cell proliferation through the reduction of insulin/IGF1 axis.

It has already been discussed that hyperinsulinemia increases cancer risk in healthy subjects and can partly explain the obesity–cancer risk association. In this perspective, in the past years, metformin has been extensively studied as a possible anticancer agent. In fact, due to its mechanism of action, metformin might interfere with carcinogenesis through direct and indirect mechanisms (Figure 1 and Figure 2).

Many preclinical studies have shown that metformin has a direct antiproliferative effect on cancer cells in vitro and in vivo; in particular, this effect seems to be related to the interference with the AMPK pathway, a central cellular key energy sensor allowing cell division, and an integrator of different regulatory inputs implicated in cell growth [48]. Evidence from epidemiological studies indicate that overweight/insulin resistant women seem to benefit more from the use of metformin in terms of BC incidence as compared to other antidiabetic drugs; these results could be related to the indirect anti-cancer effect of metformin through the reduction of systemic insulin levels and hyperglycemia [49].

In conclusion, the potential anti-cancer activity of metformin is based on two principal aspects: first, its capacity for lowering circulating insulin levels with indirect endocrine effects that may impact tumor cell proliferation; second, its direct influence on the many pro-cancer signaling pathways regulated by PI3K/AKT/mTOR, which are key drivers of BC aggressiveness.

On the basis of these observations, many clinical trials are evaluating the possible role of metformin in BC prevention and care [50]. In the past few years, epidemiologic studies reported discordant results, with a reduction in cancer incidence and mortality in some and no effect in other observations. A meta-analysis of Gandini et al. found that metformin administration was significantly associated with decreased BC incidence after adjustment for time-related biases, even though the magnitude of this effect was rather small [49].

The possible anticancer effects of metformin have also been prospectively investigated in a different setting of BC care.

A recent clinical trial showed that metformin acts on BC tumor cell proliferation differently, with a positive trend toward inhibiting proliferation only in women with insulin resistance or higher BMI [51].

In a large observational study of patients with early BC treated with pre-operative chemotherapy, the rate of pathological complete responses was significantly higher in diabetic patients treated with metformin, as compared to diabetic patients treated with other antidiabetic drugs and to non-diabetic BC patients [52]. In the adjuvant setting, a large trial on non-diabetic women with early BC comparing metformin with matching placebo in terms of disease-free survival (DFS) is currently ongoing [53].

### Metformin and Breast Cancer: Clinical Evidence from Prospective Randomized Trials

Two prospective clinical trials have explored the possible anticancer activity of metformin in BC patients in the early and in the metastatic setting. In the pre-operative setting of early BC, the possible role of metformin was evaluated in a window of opportunity (WOP) trial of 2–4 week administration between BC diagnosis and surgery has been investigated in many clinical trials [51,54,55]. In all these studies, the possible effects of metformin in inhibiting cancer cell proliferation was assessed by analyzing tumor proliferation, i.e., Ki 67, gene expression modification or reduction of insulin resistance related markers.

In particular, a randomized, phase II, double-blind, placebo-controlled trial conducted at the European Institute of Oncology, Milan, Italy, compared metformin or placebo administered for 4 weeks before surgery in women with stage I–II BC, candidate to elective surgery [56]. The primary objective was to evaluate the antiproliferative activity of metformin in terms of Ki67 modification. Overall, 200 patients were randomized: metformin was not associated with a significant reduction in cancer cell proliferation overall, however, a significant reduction was observed in insulin resistant women, identified by an HOMA Index value ≥ 2.5.

More recently, the MYME trial, a phase II, open-label, multicenter, randomized clinical study, aimed to evaluate the possible anticancer effect of metformin when added to first line chemotherapy in Her2 negative advanced BC. This study enrolled Her2 negative patients with endocrine-resistant disease, without known T2DM; assessment of HOMA index was performed in all patients at baseline, before the start of treatment. Patients were randomized to receive liposomal doxorubicin plus cyclophosphamide plus metformin 1000 mg twice daily, or chemotherapy alone. Chemotherapy was administered every 21 days for a maximum of 8 cycles, metformin was administered until disease progression. Randomization was stratified by center and HOMA index (<2.5 vs. ≥2.5), chosen as the cut-off value for insulin resistance based on the results from an Italian-based population study [57]. One hundred twenty-six patients have been enrolled. This was an interesting study, as it paid attention to anthropometric evaluations, including BMI calculation and the waist to hip ratio (WHR); in parallel, it performed host metabolism serum analyses such as glycaemia, lipidaemia, serum free fatty acid levels, triglycerides, total cholesterol, high and low density lipoprotein, hormones (insulin, C-peptide, norepinephrine, cortisol), and inflammatory markers. This is the first study to evaluate the impact of the addition of metformin to first-line chemotherapy in BC: no advantage in progression free survival (PFS) and OS was, however, observed in the combination regimen. Unexpectedly, no benefit in PFS was observed in insulin-resistant patients. This was surprising, since previous data demonstrated a significant interaction between metformin and HOMA index, especially in luminal B tumors, supporting the hypothesis of an indirect effect of metformin in insulin-resistant patients [51]. The MYME study does not confirm this hypothesis, suggesting that the potential anti-cancer effect of metformin on host metabolism could be less important in modulating response to chemotherapy. Of note, this study demonstrated a possible independent prognostic role of insulin, as insulin-resistant patients showed a significantly lower PFS compared to non-insulin-resistant women, irrespective of the use of metformin. Furthermore, in this analysis, no detrimental role of BMI and obesity was observed. From all these findings, we are able to conclude that obesity, per se, is not an independent prognostic factor in BC, whereas plasma insulin levels and insulin resistance seem to be related to BC prognosis. Interestingly, metformin demonstrated to be able to restore insulin sensibility in a subgroup of patients with basal HOMA index >2.5, without a beneficial effect in PFS and OS.

## 5. Translational Insights

The prognostic role of the insulin/IGF pathway has also been investigated on circulating tumor cells (CTCs) in BC patients. The analysis of the IGF-1R expression on CTCs of metastatic patients was first performed by de Bono et al. [58]; in their experience, they were able to detect IGF-1R expression by immunofluorescence on CTCs. Moreover, patients with high CTCs levels at baseline had high IGF-1R-positive CTCs and a more rapid disease progression. Due to the low number of CTCs the technique has been further improved to minimize cell loss during the labeling and analysis process by Pizon et al., who developed a maintrac^®^ method to avoid cell loss, omitting all enrichment steps during the preparation [59]. With this approach, they reported that 84% of BC patients harbored IGF-1R positive CTCs with a concordance between IGF-IR expression and CTCs count. Conversely, Spiliotaki et al. reported a higher expression of IGF-1R is on CTCs in patients with early BC versus metastatic disease with a progressive reduction during the transition from early to metastatic stage [60]. Overall, in this study, IGF-1R expression was detected in 79% of CTC-positive metastatic patients. Moreover, higher levels of IGF-1R positive CTCs before starting adjuvant chemotherapy were correlated with an improved prognosis in terms of DFS and OS. Recently, the TransMyme project has evaluated the prognostic role of IGF-1R expression on CTCs collected in 72 patients enrolled in the MYME study [56]. Interestingly, an increased risk of disease progression or death was observed in patients with ≥4 IGF-1R negative CTCs; this was the largest prospective observation so far, evaluating the prognostic role of IGF1-R expression on CTCs in BC patients and these results support further evaluation of the role of IGF-1R on CTCs to improve patient stratification and to implement new targeted strategies.

Another emerging issue is the cross-talk between cancer metabolism and host related immune status, due to the relationship between metabolic impairment and chronic inflammation. In particular, current research is focused on the evaluation of aberrant myelopoiesis, leading to an immunosuppressive environment, possibly due to the accumulation of myeloid-derived suppressor cells (MDSCs) that suppress adaptive immunity favoring cancer cell proliferation [31,61].

## 6. Conclusions

A growing body of evidence indicates a strong association between host metabolism and cancer. Hyperinsulinemia and obesity play important and independent roles and have been consistently associated with an adverse prognosis in many types of cancers, including BC. A possible correlation between BMI, hyperinsulinemia, metabolic dysfunction, increase of circulating inflammatory cytokines and BC risk has been reported in retrospective and prospective evidences. Metformin has been shown to interfere with carcinogenesis through direct and indirect mechanisms, supporting the hypothesis of its possible role in preventing biologically aggressive BC; however, its possible role as an anticancer agent in BC is still controversial and evidence from randomized prospective studies is lacking. The possible interplay between metabolism and the immune system in cancer patients is currently considered an emerging research field. In conclusion, obesity, metabolic syndrome, and insulin resistance are all involved in BC risk and prognosis; this effect is possibly mediated by a pro-inflammatory state, mediated by cytokines originating in fat tissue.

## Figures and Tables

**Table 1:** Insulin-like growth factor (IGF) expression (%) according to breast cancer (BC) patients’ characteristics: A higher expression is observed in patients with worse prognostic features.

BC Characteristics	IGF Expression (%)
Histological subtype	
ER+	60
TNBC	20
Tumor staging	
T1-T2	35
T3-T4	65
Lymph nodes metastasis	
Yes	60
No	40

ER: Estrogen receptor; TNBC: Triple-negative breast cancer. Adapted from [15,16].

## Data Availability

Data sharing not applicable.
